# Shaking Device for Homogeneous Dispersion of Magnetic Beads in Droplet Microfluidics

**DOI:** 10.3390/s23125399

**Published:** 2023-06-07

**Authors:** Maria Poles, Alessio Meggiolaro, Sebastian Cremaschini, Filippo Marinello, Daniele Filippi, Matteo Pierno, Giampaolo Mistura, Davide Ferraro

**Affiliations:** Department of Physics and Astronomy, University of Padua, Via Marzolo 8, 35131 Padua, Italy

**Keywords:** microfluidics, magnetic beads, sedimentation, mechanical shaking, droplet microfluidics, purification assays

## Abstract

Magnetic beads (or particles) having a size between 1 and 5 µm are largely used in many biochemical assays devoted to both purification and quantification of cells, nucleic acids, or proteins. Unfortunately, the use of these beads within microfluidic devices suffers from natural precipitation because of their size and density. The strategies applied thus far to cells or polymeric particles cannot be extended to magnetic beads, mainly due to their magnetization and their higher densities. We report an effective shaking device capable of preventing the sedimentation of beads that are stored in a custom PCR tube. After the characterization of the operating principle, the device is validated for magnetic beads in droplets, leading to an equal distribution between the droplets, barely affecting their generation.

## 1. Introduction

Microfluidics is experiencing large diffusion in many different biological applications, especially in the development of a new generation of biomedical instruments devoted to the diagnosis of various types of cancer and degenerative diseases [1,2]. As a matter of fact, the major microfluidic goal is the integration of conventional protocols, typically performed in large and equipped laboratories, within the so-called lab-on-a-chip devices capable of handling, detecting, and analyzing various biological contents, such as proteins, nucleic acids, and cells [3,4].

During recent decades, the use of magnetic beads has experienced a large diffusion in both research and clinical diagnostics, especially for the purification, isolation, and quantification of rare biomarkers, such as cells, nucleic acids, proteins, or extracellular vesicles [5,6,7,8,9,10,11,12,13]. In detail, magnetic beads, whose average sizes are between 1 and 5 µm, are functionalized to increase the affinity of the target solutes that are dispersed in a liquid mixture. In this way, the separation of the desired components from the supernatant can be easily achieved using an external magnet [14,15,16]. Therefore, their use is constantly increasing due to the simplicity of the proposed protocols, the variety of applicable surface chemistry, and their high surface-to-volume ratio compared to flat surfaces [17]. Furthermore, they are compatible with many commonly performed analytical methods, such as flow cytometry [18], ELISA [19], or electrochemical detection [20], and have already been used in various clinical applications [21], even to monitor biomarkers before and after surgery [22].

Despite their diffusion in various biomedical and diagnostic applications, the current integration of magnetic beads in microfluidic devices is still poorly explored, mainly due to handling limitations. One of the most critical aspects that prevents their use is their rapid sedimentation, due to their sizes and densities. In fact, the sedimentation speed v is driven by Stokes law $v=d^{2}\left(\rho_{o}-\rho_{f}\right)g/\left(18\;\mu_{f}\right)$, where d and $\rho_{o}$ are, respectively, the diameter and density of the single bead, $\rho_{f}$ and $\mu_{f}$ the density and viscosity of the surrounding liquid, and g is the acceleration of gravity [23,24,25]. For example, considering a magnetic bead of 3 µm ($\rho_{d}$ ~ 1.8 g/cm^3^) dispersed in water ($\mu_{f}$ ~ 1 cP), its sedimentation speed is about 0.25 mm/min, leading to a precipitation length of 1 mm in less than 5 min. Therefore, in a typical microfluidic experiment that can last several minutes or even hours, magnetic beads tend to sediment within the storage reservoir (e.g., syringe, vial, well of a microtiter plate) before they enter the microfluidic circuit. This leads to two sources of problems: (i) loss of part of the beads, which are usually expensive, and (ii) infusion of non-homogeneous beads that can induce biased results, especially if they are used for the quantification of rare tumor cells [26,27] or for immunoagglutination purposes [28,29]. Furthermore, in the case of beads that must be encaspulated within continuously generated droplets [17,30,31,32,33], their concentration can strongly vary between the first and last generated droplets, as shown below.

Usually, in small-scale laboratory studies, the bead sedimentation problem is overcome by manually shaking the reservoir before the experiment. However, automated and user-independent strategies are currently not available. In the case of non-magnetic objects still prone to sedimentation, such as polimeric particles or cells, automated magnetic stirring can be applied [34,35,36]; however, this approach cannot be used for magnetic beads because they would easily aggregate. Alternatively, other approaches that do not involve magnetic components have been introduced to date, based on the syncronization of the precipitation of magnetic beads with the generation of droplets [37]. In detail, this approach has proven to be effective for single-bead encapsulation in droplets; nevertheless, the concentration of beads needs to be adjusted according to the droplet generation rate, limiting the possible applications. Rotating syringe pumps have also been developed to continuously resuspend polystyrene beads [38], but this approach has not been validated using magnetic beads that have a double density compared to polymeric particles.

In this work, we present a novel and effective shaking device to prevent magnetic bead sedimentation. The device is composed of a custom 3D-printed support that holds a small vibrating motor coupled to a custom PCR tube. First, we describe the realization steps and assess its performance. Then, to show its effectiveness in real applications, we test the shaking device combined with a microfluidic chip presenting a T-junction design commonly used for droplet generation in many clinical assays that require the handling of magnetic beads (e.g., nucleic acid purification, single cell analysis, immunoassay, etc.) [21]. These tests clearly show that the beads are equally distributed between the droplets when the shaking device is activated; otherwise, they accumulate mostly within the last droplets. Finally, the specifically designed PCR tube allows the use of the entire beads’ sample, avoiding waste.

## 2. Materials and Methods

### 2.1. Microfabrication of the Shaking Device

**Figure 1:**
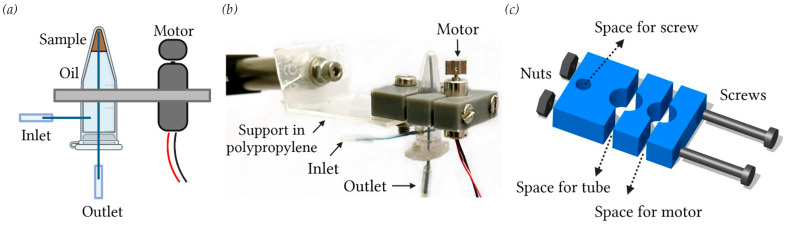
(**a**) Scheme and (**b**) picture of the vibrating device that prevents bead sedimentation inside the custom PCR tube turned upside down. (**b**) The 3D-printed components (grey parts) ensure an integral motion between the small vibrating motor and the tube, whereas the polypropylene support allows decoupling this motion with the setup. (**c**) Schematic view of the 3D-printed parts holding both the motor and the tube.

The shaking device shown in Figure 1a,b was based on a small vibrating motor (Precision Mini Drives, NFP-P1015, Shenzhen, China) coupled to a tube containing the magnetic bead suspension. The two components were tied together by three 3D-printed parts (see Figure 1c) made by a stereolithographic 3D printer (Form 3, by Formlabs, Somerville, MA, USA), using Grey V4 resin (by Formlabs, Somerville, MA, USA). The latter was chosen because its large Young modulus ($E_{Y}$ = 2.2 GPa) prevented any damping effect between the motor and the tube; the two parts could thus move together as a single body (see Video S1, Supplementary Materials). In contrast, a polypropylene support (see Figure 1b) was used to isolate the shaking device; in detail, the polypropylene being easy deformable ($E_{Y}$ = 1.4 KPa), it allowed the movement of the shaking device to be decoupled from the rest of the setup. Therefore, upon activation, the small vibrating motor transferred its motion to the tube and, in turn, to the contained liquids that could be continuously shaken. Source files (STL format) for the 3D-printed parts are available in the Supplementary Materials (see Note S1). Notably, the PCR tube was installed upside down with respect to its conventional use; this configuration was chosen because its cross section decreased close to the bottom edge. In this way, the contained liquid was confined in a small volume, and the outlet capillary ensured that the entire aqueous phase was collected before the oil (see Figure 1a), without sample or bead losses.

### 2.2. Syringes and Tubes for Liquid Storage

Two glass syringes (1 mL, by SGE, Ringwood, Victoria, Australia) mounted on two independent syringe pumps (PHD 2000, by Harvard apparatus, Holliston, MA, USA) were used to control droplet generation and motion. The use of a syringe for the infusion of the bead suspension presented the drawback that part of the sample stored inside could not be completely flown into the microfluidic device, remaining in the syringe itself or along the capillary used for inlet connections to the microfluidic device. To avoid this problem, a conventional 0.5 mL PCR tube (by ThermoFisher Scientific, Waltham, MA, USA) was customized by fixing two capillaries (PEEK, inner/outer diameter 0.25/0.79 mm, by IDEX, Oak Harbor, WA, USA) in two drilled holes: one on the side and the other on the cap, representing the inlet and the outlet, respectively (see Figure 1a,b). The tube was then completely filled with the magnetic bead suspension and the fluorinated oil; the oil being denser than the aqueous suspension (1.86 g/cm^3^ and 1.05 g/cm^3^, respectively). Once the tube was turned upside down with respect to conventional use, the beads immediately moved above the oil (see Figure 1). Thus, by pushing the oil from the inlet with a prefilled syringe, the bead suspension was forced to exit the tube from the outlet capillary. Notably, in this configuration, the sample could be entirely infused without losses, and then the remaining oil was flown out of the tube. By turning the PCR tube upside down, the collection of the contained liquid by the outlet capillary that was fixed inside was more accurate. Unlike other strategies based on prefilling the sample within a long capillary [39], this approach did not present limitations in terms of sample quantity.

During microfluidic experiments, PEEK capillaries were connected to PTFE capillaries (inner/outer diameter: 0.3/0.6 mm, by Sigma Aldrich, Darmstadt, Germany) through a silicone joint (inner/outer 0.5/2.5 mm, by Deutsch & Neumann, Hennigsdorf, Germany).

### 2.3. Reagent Preparation

Aqueous suspensions of magnetic beads with a mean diameter of 2.8 µm (Dynabeads M-270 carboxylic acid, by Invitrogen, Waltham, MA, USA) were prepared at different concentrations (between 10^8^ and 10^9^ beads/mL). Before use, the magnetic beads were washed twice and stored in PBS (1× Phosphate buffered saline) solution. Fluorinated oil (FC40, by 3M, Saint Paul, MN, USA) mixed with 2% surfactant (Krytox 157 FSH, by Chemours, Wilmington, DE, USA) was used for droplet generation. The surfactant decreased the surface tension between the oil and the aqueous phase, allowing the generation of droplets and promoting their stability during motion [40,41].

### 2.4. Microfluidic Device Fabrication

The double replica molding technique was employed for the fabrication of the microfluidic device, from a brass mold realized by micro-milling (by Minitech Corp., Norcross, GA, USA). In detail, polydimethylsiloxane (PDMS, Sylgard 184, by Dow Corning, Midland, MI, USA) allowed replicating the initial brass mold, and the resulting part was then used as mold as well [42]. Silanization in vapor phase (1H,1H,2H,2H-Perfluorooctyltriethoxysilane, by Alfa Aesar, Kandel, Germany) was applied to the first PDMS replica to prevent its adhesion during the second molding step [43]. The resulting microfluidic device was closed with another PDMS part by oxygen plasma. The microfluidic circuit consisted of a T-junction, having a channel cross section of 200 µm and 300 µm (height and width, respectively). After their generation, the droplets were flown out of the device and stored in a PTFE capillary (inner/outer diameter: 0.5/1.0 mm, by Sigma Aldrich) for observation and quantification of magnetic beads. An iron tip (size of 1.8 mm) magnetized by a permanent cylindric magnet (diameter 2 cm, height 1.5 cm, by Supermagnete, Gottmadingen, Germany) was placed close to the capillary where droplets flowed, allowing the accumulation of the contained beads.

### 2.5. Optical Setup for the Characterization and Validation of the Shaking Device

To acquire image sequences of the activation of the shaking device, a fast camera (VEO-E 310L, by Phantom, Wayne, NJ, USA) was used, coupled to a macro zoom lens (LiNOS MeVis C 50 mm/f1.8, by Baumer, Frauenfeld, Switzerland). Instead, a CMOS camera (Basler acA1300–200 µm) coupled with a telecentric lens (coaxial 2 × 110) was used to evaluate the distribution inside the droplets after their formation within the microfluidic device and storage within the PTFE capillary. A white LED backlight was required to illuminate the capillary.

## 3. Results and Discussion

The performance of the shaking device in preventing the sedimentation of the beads was validated by (i) measuring the bead sedimentation rate within the tube and (ii) by evaluating the magnetic bead distribution inside the droplets. In this second case, we also characterized the possible vibration effects on the droplet generation, in terms of the droplet size polydispersity.

### 3.1. Sedimentation of Beads in the Tube

**Figure 2:**
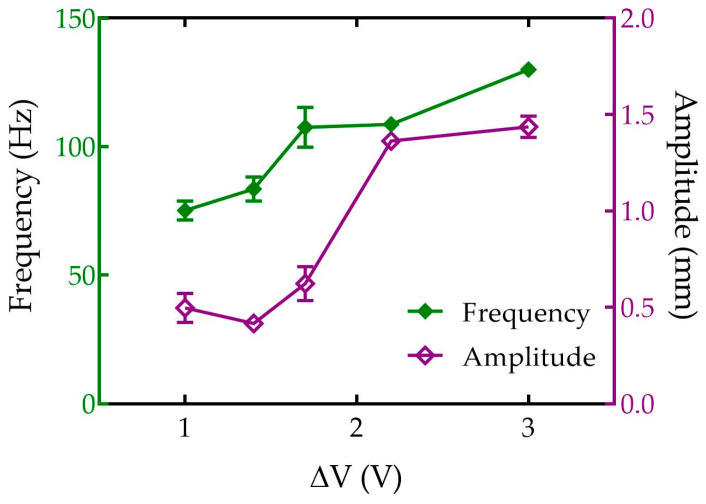
Characterization of the vibration frequency (green) and amplitude (purple) of the shaking device (see Figure 1) for different voltages (ΔV) applied to the motor. Data are the average results of three repeated measurements, and error bars are the corresponding standard deviations.

The shaking device was first characterized in terms of amplitude and frequency of vibration for different applied voltages (ΔV) to the motor, with the PCR tube filled with Phosphate Buffer Saline solution (PBS). Image sequences (see Video S1, Supplementary Materials) acquired by the fast camera (3000 fps) were analyzed by ImageJ software, showing that both frequency and amplitude increased with V, as reported in Figure 2 (see also Table S1, Supplementary Materials). Although the oscillation frequency increased almost linearly with V, its amplitude appeared constant for applied voltages lower than 2 V and drastically increased for higher values, sometimes leading to damage to the PCR tube. For that, the voltage applied during the sedimentation experiments was kept at 1.7 V. The sharp increase in the amplitude observed above 1.7 V was due to a mechanical resonance of the entire shaking device (see Note S2, Supporting Information).

**Figure 3:**
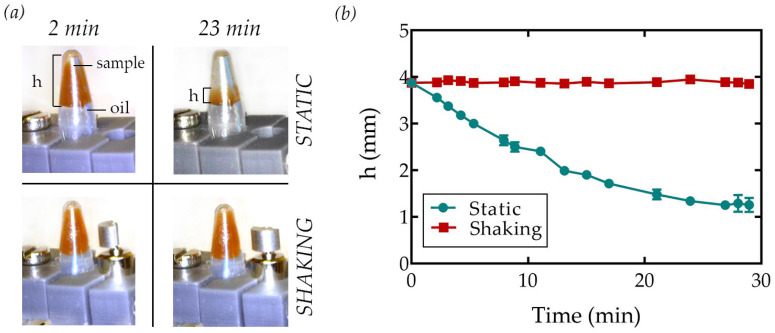
(**a**) Picture of the shaking device under static (**top**) and shaking (**bottom**) conditions at the beginning of the experiment (*t* = 2 min) and after 23 min. The presence of the magnetic beads is clearly indicated by the brown regions within the tip of the inverted PCR tube. (**b**) Height (*h*) of the bead suspension measured from the beads–oil interface under static (green) and shaking (red) conditions evaluated in time. Error bars represent the standard deviation of the average of three independent measurements. If not visible, the error bars are smaller than the size of the datapoints.

Magnetic bead sedimentation within the tube was then monitored for 30 min. First, 100 µL of magnetic bead suspension was inserted into the tube, which was then completely filled with fluorinated oil, taking care not to trap air. Parallel experiments were performed to observe the sedimentation of beads in the presence (shaking) and absence (static) of vibration. The difference between static and shaking conditions was also evident to naked eyes from the pictures reported in Figure 3a. The sedimentation rate (see Figure 3b) was quantified by measuring the distance (*h*) between the beads–oil interface and the upper tip of the PCR tube (see Figure 3a). The graph shows that the beads precipitated almost completely after 30 min in the static case, whereas sedimentation was not observed under shaking conditions, proving the functionality of the device. Furthermore, as shown in Note S3 of the Supplementary Information, the shaking device prevented beads sedimentation even for a longer time (tested up to 75 min).

### 3.2. Encaspulation of Beads within Droplets and Droplet Size Distribution

**Figure 4:**
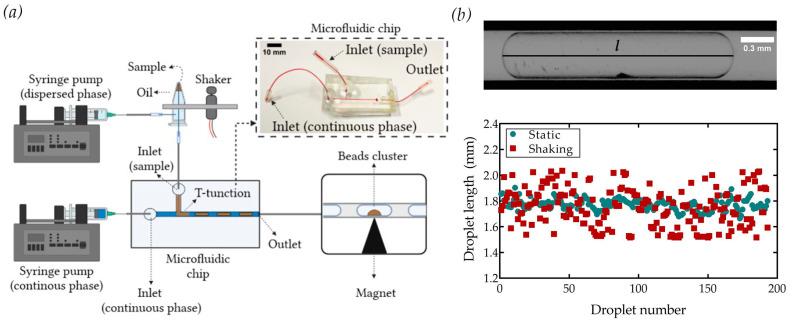
(**a**) Scheme of the microfluidic setup for the droplet generation and encapsulation of the magnetic beads, with a picture of the PDMS microfluidic device. (**b**) Scatter plot of the droplet length *l* generated in the static (green) and shaking (red) modes.

To evaluate the applicability of the shaking device in homogenously distributing magnetic beads over a sequence of generated droplets, the experimental setup reported in Figure 4a was assembled. Here, a conventional T-junction was used for droplet generation, and the liquids were handled by two independent syringes. In particular, the syringe controlling the dispersed phase (droplets) was connected to the inlet of the PCR tube mounted in the shaking device and then to the microfluidic chip by a capillary having a length of about 10 cm. Generated droplets were horizontally flown out the chip toward a PTFE capillary for observation. This configuration prevented droplet breakup at the outlet [31].

Consistent with the results of Figure 3, the shaking device must have been continuously activated during the droplet generation process to prevent bead sedimentation. Therefore, preliminary experiments were performed to assess the eventual influence of vibration on the droplet size: parallel tests were performed under static and shaking conditions applying the same flow rates (5.5 µL/min and 11 µL/min for the continuous and dispersed phases, respectively). Figure 4b reports the size distributions of the droplets generated without (green dot) and with vibration (red dot). Here, no variations were observed in terms of average droplet size (static: 1.77 ± 0.05 mm, shaking: 1.75 ± 0.14 mm), whereas a larger polydispersity of the droplets was observed with the shaking configuration (static: 3%, shaking: 8%). Similar results of polydispersity were found for lower droplet volumes (see Note S4, Supplementary Information).

**Figure 5:**
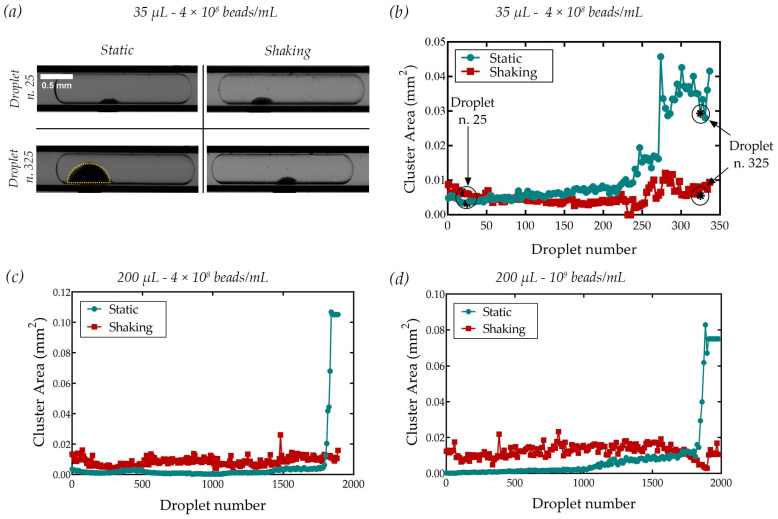
(**a**) Picture of one of the initial (top) and final (bottom) generated droplets with the bead cluster formed by magnetic attraction. (**b**–**d**) Area of bead clusters measured under static and shaking conditions generated for suspension at different concentrations and volumes: (**b**) 4 × 10^8^ beads/mL of 35 μL, (**c**) 4 × 10^8^ beads/mL of 200 μL (**c**), and (**d**) 10^9^ beads/mL of 200 μL.

Finally, to evaluate the magnetic bead encapsulation within the generated droplets, the latter was transported in correspondence with a magnetized iron tip, leading to clustering of beads (see Figure 5a). In particular, although this configuration could have been used for the extraction of beads from the droplet [16,31,44], it should have been considered that the magnetic force could have been adjusted to induce cluster formation without achieving their extraction [45]. For this reason, the magnetic force was tuned to be lower than the capillary force, typically ranging between 1 µN and 10 µN for this dimension [21]. The image sequences were analyzed by measuring the cluster size using ImageJ software: the cluster area was approximated as a hemi-ellipse, as schematically shown in Figure 5a. The cluster area (expressed in mm^2^ in the graphs) was directly related to the quantity of beads in each droplet, as already applied for the immunoagglutination assay [29]. Figure 5b reports the results varying the volume of the sample (35 µL and 200 µL) and the bead concentration (4 × 10^8^ beads/mL and 4 × 10^9^ beads/mL). These values were chosen because they were typically used in many applications, such as immunoassays, mRNA and DNA purification, or extracellular vesicle isolation [17,46,47]. Figure 5 shows that, in all cases, activation of the shaking device promoted a good homogeneity of the bead distribution along the droplet train. In fact, under static conditions, most of the droplets contained a low number of beads, whereas the last ones had a tremendously higher quantity. This non-uniform distribution of magnetic beads over the droplets was even more evident by increasing the starting sample volume, and thus the number of droplets. The same trend was also found using a higher bead concentration (10^9^ beads/mL).

## 4. Conclusions

We realized a shaking device that was effective for continuously homogenizing a suspension of magnetic beads within a storage tube. In this manner, the solution is ready to be used before and, more importantly, during automated microfluidic experiments that require a uniform distribution of beads for subsequent analysis purposes. The feasibility of our approach was effectively shown for a typical size of the magnetic beads (2.8 µm) at different initial concentrations and volumes. Furthermore, the shaking device could work for long-term experiments (tested up to 75 min). Finally, the inverted orientation of the PCR tube prevented the loss of sample, which, being collected from the narrow part of the tube, was completely injected within the microfluidic channels.

The use of magnetic beads in droplet microfluidics is widely used in many biological applications [21] (e.g., agglutination assays [29,48,49], nucleic acid isolation and analysis [16,31], and protein investigation [30,50]). In this context, sedimentation is typically prevented by manually shaking the reservoir before or during the experiment, being highly operator-dependent. We believe that the proposed strategy can be easily implemented in many existing devices in order to improve their automation and industrialization aspects. Additionally, the shaking holder can allow for a better evaluation of time-dependent results, preventing bias in terms of quantification of target molecules or rare cells [26,29].

Magnetic beads are also employed in monophasic microfluidic devices for various biological procedures [51], such as cell sorting [52,53] and the purification of extracellular vesicles [25,54]. Therefore, even though the shaking device has been validated for the bead encapsulation in the droplets, it can also be successfully implemented in monophasic microfluidic systems.

## Data Availability

Not applicable.

## Supplementary Materials

The following supporting information can be downloaded at: https://www.mdpi.com/article/10.3390/s23125399/s1, Video S1: Shaking device; Note S1: 3D Design of the shaking device; Table S1: Shaking device characterization data; Note S2: Mass contribution to shaking device oscillation; Figure S1: Vibration amplitude of the shaking device for different voltages (ΔV) applied to the motor without the PCR tube installed in the device, and with the PCR tube filled with PBS solution and iron powder; Note S3: Magnetic bead sedimentation during shaking device activation; Figure S2: Pictures of the shaking device under shaking condition after 2 min, 30 min, 50 min and 75 min; Note S4: Droplet generation during shaking device activation. Figure S3: Plots of the droplet length generated in the static and shaking modes with an average volume of (a) 56 nL and (b) 28 nL.
