# Supplementary material for: Shaking Device for Homogeneous Dispersion of Magnetic Beads in Droplet Microfluidics
Source: Sensors (Basel). 2023 Jun 7;23(12):5399. doi: 10.3390/s23125399 (PMC10304097; doi:10.3390/s23125399)
Supplement: Supplementary file 1 [file sensors-23-05399-s001.zip › Poles_Sensors_SupplMat.pdf]

Supplementary Information

Shaking device for homogeneous dispersion of magnetic beads in droplet microfluidics

Maria Poles [1,†], Alessio Meggiolaro [1,†], Sebastian Cremaschini [1], Filippo Marinello [1], Daniele Filippi [1], Matteo Pierno [1], Giampaolo Mistura [1], and Davide Ferraro [1,*]

[1] Department of Physics and Astronomy, University of Padua, Via Marzolo 8, 35131 Padua, Italy; maria.poles@studenti.unipd.it (M.P.); alessio.meggiolaro@studenti.unipd.it (A.M.);
† These authors contributed equally to this work
* Correspondence: davide.ferraro@unipd.it

Video S1: Shaking device

Video of the shaking device acquired by the fast camera at 3000 fps and slowed down 150 times. When activated, the small vibrating motor transfers the motion to the PCR tube, which causes its contents to shake.

Note S1: 3D Design of the shaking device (*.stl)

Left-part.stl, Center-part.stl, Right-part.stl are the files that are used to print the shaking device reported in Figure 1 (main paper). The different parts are printed and assembled together to block the small vibrating motor and the PCR tube. The files can be used directly for 3D printing.

Supplementary Table S1: Shaking device characterization data

The amplitude and frequency of vibration of the shaking device are characterized as a function of the different applied voltages (ΔV). Data from Figure 2 (main paper) are reported in Table S1, showing that both frequency and amplitude increase with voltage. The data reported are the average results of three repeated measurements, together with the related standard deviation.

**Voltage (ΔV)**	**Amplitude (mm)**	**Frequency (Hz)**
1 V	0.50 ± 0.08	75 ± 2
1.4 V	0.41 ± 0.04	83 ± 2
1.7 V	0.62 ± 0.09	107 ± 4
2.2 V	1.36 ± 0.03	109 ± 1
3 V	1.44 ± 0.06	130 ± 1

Note S2: Mass contribution to shaking device oscillation

The vibration frequency of the shaking device increases with the applied different voltages (ΔV) due to the mode of operation of the small motor. The amplitude of the vibration is characterized for different voltages (ΔV) applied to the motor under three different conditions: i) without the PCR tube installed and with the tube filled ii) with PBS solution and iii) with an iron powder (mass of 5.5 g). Image sequences, acquired by the fast camera (3000 fps), are analyzed by Image J software. Amplitude was evaluated as the maximum variance of the shaking device from its starting position. The results are reported in Figure S1, showing different trends for the three conditions: the empty system shows an almost linear increase (grey), while in the other two cases the amplitude increases for ΔV> 1.7V; however, in the presence of the iron powder (blue), the amplitude decreases for ΔV> 2.2V. This may be due to a resonance phenomenon. Therefore, it is recommended to characterize the amplitude variation in shaking system when changing the mass or the shape of the tube inserted in the device.

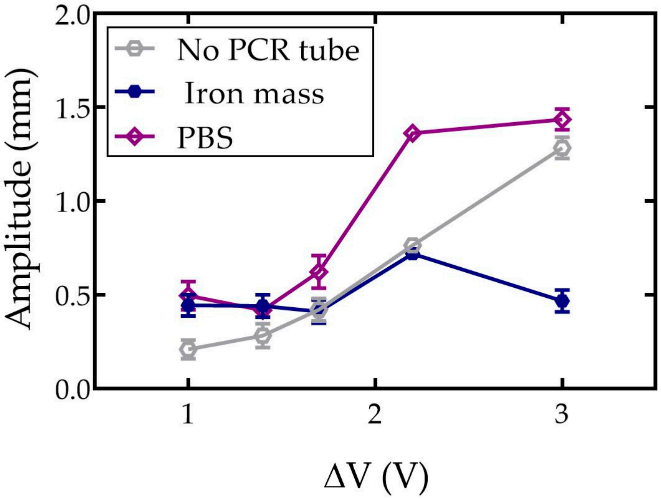

Supplementary Figure S1: Vibration amplitude of the shaking device for different voltages (ΔV) applied to the motor without the PCR tube installed in the device (grey), and with the PCR tube filled with PBS solution (purple) and iron powder (blue). Data of PBS solution (purple) correspond to data in the Figure 2 of the main paper. Data are the average results of three repeated measurements and error bars are the corresponding standard deviation.

Note S3: Magnetic bead sedimentation during shaking device activation

The continuous suspension of the bead solution during the activation of the shaking device is characterized for 75 minutes and, as reported in Figure S2, no sedimentation is visible.

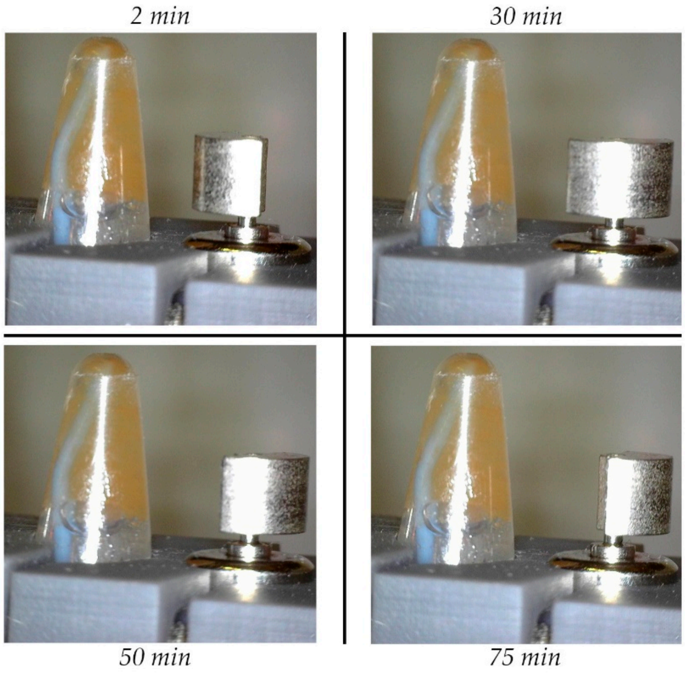

Supplementary Figure S2: Pictures of the shaking device under shaking condition after 2 minutes (up, left), 30 minutes (up, right), 50 minutes (bottom, left) and 75 minutes (bottom, right). The brown region in the PCR tube indicates the presence of beads in suspension.

Note S4: Droplet generation during the shaking device activation

The influence of vibration on droplet size is tested for different flow rates combinations: 5.5 (25) μL/min and 11 (2) μL/min are used for the continuous and dispersed phases, respectively, to produce droplets of 56 (28) nL. Figure 3 reports the scatter plot of the length of the droplets as a function of their number. In detail, Figure S3a shows that, for droplets of 56 nL, no significant variations are observed in terms of the average droplet size (static: 0.93 ± 0.02 mm, shaking: 0.93 ± 0.05 mm), while a larger polydispersity of the droplets is observed with the shaking configuration (static: 2%, shaking: 5%). Then, generating droplets of 28 nL (see Figure S3b), their average size remains unvaried (static: 0.44 ± 0.05 mm, shaking: 0.49 ± 0.06 mm) and a similar polydispersity is observed between the two conditions (static: 11%, shaking: 12%).

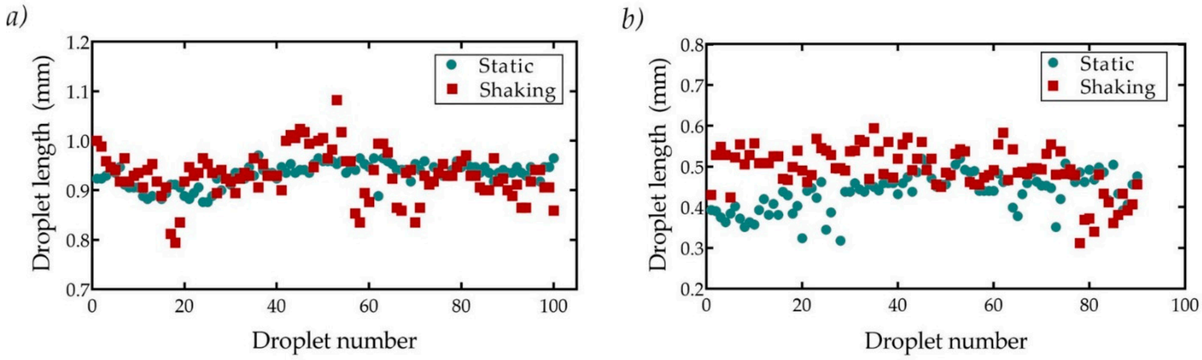

Figure S3: Plots of the droplet length generated in the static (green) and shaking (red) modes with an average volume of a) 56 nL and b) 28 nL.
